# Contributing factors and clinical usefulness of spot urine glucose-to-creatinine ratio by sodium glucose cotransporter 2 inhibitors: a cross-sectional study

**DOI:** 10.1007/s40620-025-02305-6

**Published:** 2025-05-19

**Authors:** Kojiro Nagai, Keisuke Morita, Ken Matsuo, Satoshi Tanaka

**Affiliations:** Department of Nephrology, Shizuoka General Hospital, 4-27-1, Kita Ando, Aoi-ku, Shizuoka, 420-8527 Japan

**Keywords:** Sodium glucose cotransporter 2 inhibitor, Urinary glucose excretion, Diabetes, Chronic kidney disease

Sodium glucose cotransporter 2 (SGLT2) is expressed on the luminal side of the proximal tubule and reabsorbs the glucose filtered by the glomeruli [1]. Clinical trials have demonstrated renal and cardiovascular protective effects of SGLT2 inhibitors [2–4]. However, evidence is lacking for these effects in patients with advanced chronic kidney disease (CKD) stages G4-5 [5, 6].

Multiple mechanisms underlie the protective effects of SGLT2 inhibitors, primarily through the inhibition of renal glucose reabsorption. Assessing urinary glucose excretion is clinically important as it could help predict the benefits of SGLT2 inhibitors.

Pharmacological studies on SGLT2 inhibitors have established that renal impairment and diabetic status affect urinary glucose excretion. Yet, other influential factors remain unexplored, and there are scant urinary glucose excretion data from actual patients [7].

Spot urine tests can conveniently estimate the 24 h excretion rates of substances. It is essential to determine whether the spot urine glucose-to-creatinine ratio can predict 24 h urinary glucose excretion.

This cross-sectional study included 284 outpatients with CKD who visited the Department of Nephrology at Shizuoka General Hospital between April, 2022 to December, 2022. Fifty-three patients were excluded due to a lack of laboratory data, and an additional five were excluded for noncompliance with treatment that was discovered by interview survey. Ultimately, 226 patients (mean ± SD: 66.4 ± 13.7 years; 43 females; 168 diabetics) were enrolled. Blood and urine samples were collected at any time for biochemical analysis. Creatinine-based estimated glomerular filtration rate (eGFRcre) was calculated using the formula: eGFRcre (mL/min/1.73 m^2^) = 194 × serum creatinine^−1.094^ × age^−0.287^ × 0.739 (if female) [8]. This group included 100 (44.2%) and 98 (43.4%) patients with CKD stages G4-5 and stage G3, respectively. The mean eGFRcre was 37.4 ± 22.3 mL/min/1.73m^2^. Ninety-three (41.2%) and fifty-eight (25.7%) patients suffered from diabetic kidney disease and hypertensive nephrosclerosis, respectively. The main prescriptions were 10 mg of dapagliflozin (*n* = 69 (30.5%)) and empagliflozin (51 (22.6%)) once daily. Among the 226 patients, 39 completed a 24 h urine collection.

Statistical analyses were conducted using SPSS for Windows (version 13.0; SPSS, Inc., Chicago, IL, USA). The factors related to spot urine glucose-to-creatinine ratio were initially assessed using single regression analysis. Variables considered included age, serum albumin level, eGFRcre, blood glucose, HbA1c, sodium, potassium, chloride, calcium, urine protein-to-creatinine ratio, fractional excretion of urea nitrogen, and creatinine-corrected urinary tubular injury markers such as α1-microglobulin (MG)/Cre (creatinine corrected), β2MG/Cre, NAG/Cre, NGAL/Cre, and L-FABP/Cre. Multiple regression analysis was performed using variables that showed *P* of less than 0.1 in single regression analysis. Correlation was determined by Pearson’s correlation coefficient test. Low urinary glucose excretion was tentatively defined as a urine glucose-to-creatinine ratio less than 30 g/gCr, based on a predicted value from our study’s single regression analysis, where the spot urine glucose-to-creatinine ratio for patients with an eGFRcre of 30 ml/min/1.73m^2^ was 25.9 g/gCr. Statistical significance was defined as *P* less than 0.05.

Regression analysis was performed in all patients. Age, serum albumin level, eGFRcre, blood glucose, HbA1c, potassium, chloride, calcium, fractional excretion of urea nitrogen, β2MG/Cre and α1MG/Cre were identified as possible factors. Correlations are shown in Fig. 1a. The independent factors selected for spot urine glucose-to-creatinine ratio were eGFRcre, blood glucose, HbA1c and fractional excretion of urea nitrogen (Fig. 1b). Adjusted R^2^ was 0.618.

**Fig. 1:**
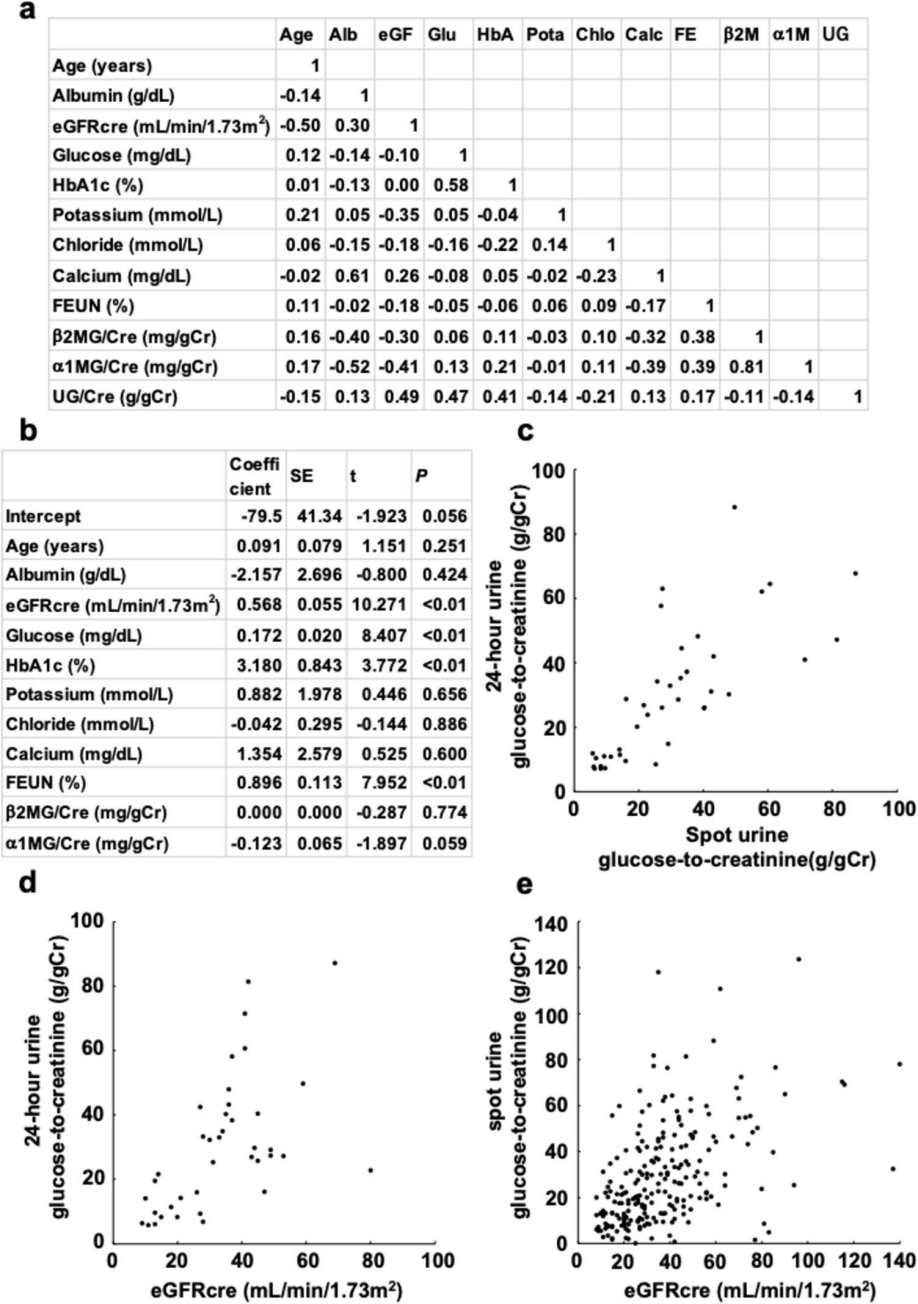
**a** Correlations between possible factors related to the spot urine glucose-to-creatinine ratio in all patients (*n* = 226). *Alb* albumin. *eGF, eGFRcre* creatinine-based estimated glomerular filtration rate. *Glu* glucose. *HbA* HbA1c. *Pota* potassium. *Chlo* chloride. *Calc* calcium. *FE, FEUN* fractional excretion of urea nitrogen. *β2M, β2MG/Cre* β2-microglobulin (creatinine corrected). *α1M, α1MG/Cre* α1-microglobulin (creatinine corrected). *UG, UG/Cre* spot urine glucose-to-creatinine ratio. **b** Significant independent factors for spot urine glucose-to-creatinine ratio in all patients (*n* = 226). *eGFRcre* creatinine-based estimated glomerular filtration rate. *FEUN* fractional excretion of urea nitrogen. *β2MG/Cre* β2-microglobulin (creatinine corrected). *α1MG/Cre* α1-microglobulin (creatinine corrected). *SE* standard error. **c** Scatter plots of 24 h urine and spot urine glucose-to-creatinine ratio (*n* = 39). **d** Scatter plots of 24 h urine glucose-to-creatinine ratio and estimated GFR (*n* = 39). eGFRcre: creatinine-based estimated glomerular filtration rate. **e** Scatter plots of spot urine glucose-to-creatinine ratio and estimated GFR (*n* = 226). *eGFRcre* creatinine-based estimated glomerular filtration rate

To investigate the pure effect of SGLT2 inhibitors on urinary glucose excretion, we analyzed 137 patients whose dipstick test for glucose before initiating SGLT2 inhibitor therapy was negative at least twice consecutively. Other sub-analyses were conducted on 69 patients taking 10 mg of dapagliflozin, and on 100 patients with CKD stages G4-5. The independent determinants of all sub-analyses were the same as those in all patients.

We compared spot urine glucose-to-creatinine ratio with 24 h urine glucose-to-creatinine ratio. Among the 39 patients who provided 24 h urine samples, only 23 (59.0%) met the 70% accuracy threshold in predicting 24 h urine glucose-to-creatinine ratio. However, among 22 patients with a spot urine glucose-to-creatinine ratio less than 30 g/gCr, 19 had a 24 h urine glucose-to-creatinine ratio less than 30 g/gCr, resulting in a positive predictive value of 86.4% (Fig. 1c).

The 24‐hour urine glucose-to-creatinine ratio varied from 5.7 to 42.5 in patients with CKD stages G4-5 and from 16.1 to 87.1 in patients with CKD stages G1-3 (Fig. 1d). Similarly, the spot urine glucose-to-creatinine ratio ranged widely (Fig. 1e).

In this study, we provide data on urinary glucose excretion from patients with advanced CKD treated with SGLT2 inhibitors, which were found to vary widely. Potential independent factors influencing spot urine glucose-to-creatinine ratio in those patients included blood glucose, kidney function, and volume status, but not tubulointerstitial injury markers. Spot urine glucose-to-creatinine ratio may be useful in identifying patients with low urinary glucose excretion due to SGLT2 inhibitors.

Previous research has shown that urinary glucose excretion by SGLT2 inhibitors is contingent upon the amount of glucose filtered into the tubule. Despite variations in renal function, there was a consistent inhibition (30–50%) of glucose reabsorption [7]. Our findings align with these studies. The relationship between urinary glucose excretion and fractional excretion of urea nitrogen is consistent with reports of a low incidence of volume depletion-related adverse events in clinical trials of SGLT2 inhibitors [2–4]. Surrogate markers of proximal tubule damage have no impact on urinary glucose excretion, suggesting that glucose reabsorption occurs irrespective of renal disease.

Spot urine glucose-to-creatinine ratio may be a useful predictor for identifying patients with low urinary glucose excretion. Notably, many patients with low urine glucose-to-creatinine ratio were observed even among those with CKD stages G1-3 (Fig. 1d, e). Spot urine glucose-to-creatinine ratio could serve as a screening test to estimate the impact of SGLT2 inhibitors and potentially reduce polypharmacy, and to avoid SGLT2 inhibitor-related side effects [9].

Further studies on the outcomes and complications in patients taking SGLT2 inhibitors in relation to urinary glucose excretion would allow us to ascertain the clinical role of urinary glucose excretion.

## Data Availability

The datasets analyzed are available from the corresponding author on reasonable request.
